# Caudal foot placement superior to toe elevation for navicular palmaroproximal‐palmarodistal‐oblique image quality

**DOI:** 10.1111/evj.13563

**Published:** 2022-02-15

**Authors:** Manon W. J. Peeters, Jasmine J. Thursby, Hannah E. Watson, Dagmar Berner

**Affiliations:** ^1^ Department of Clinical Science and Services Equine Referral Hospital Royal Veterinary College University of London Hatfield Hertfordshire UK; ^2^ Present address: Paardenpraktijk de Peelkant Wethouder Lindersstraat 143 Wilbertoord The Netherlands

**Keywords:** diagnostic imaging, horse, lameness, musculoskeletal

## Abstract

**Background:**

Palmaroproximal‐palmarodistal oblique (PaPr‐PaDiO) radiographs are regularly obtained for a full evaluation of the navicular bone (NB). Despite their routine use, different acquisition techniques are described.

**Objectives:**

To determine optimal foot placement and beam angle for obtaining PaPr‐PaDiO views.

**Study design:**

In vitro experiment.

**Methods:**

A convenience sample of 26 disarticulated forelimbs were placed in six different positions using a leg press to mimic the weight‐bearing position. In each position, navicular PaPr‐PaDiO images were obtained with eight different beam angles. The resulting 1248 radiographs were graded for their diagnostic quality and the compacta spongiosa demarcation of the NB.

**Results:**

Diagnostic quality and compacta‐spongiosa demarcation was graded higher for feet positioned caudally and angle between 40° and 45°. Elevation of the toe significantly decreased the NB palmar border angle (elevated mean: 40.66, SD: 4.46, non‐elevated mean: 42.06, SD: 4.70) (*P* < .01), but seemed to have no obvious positive influence on radiographs.

**Main limitations:**

Using disarticulated legs could only mimic positions but, using a press, weight‐bearing positions were replicated as closely as possible. The use of a convenience sample makes the results of the study exploratory only.

**Conclusions:**

Caudal foot placement seems to improve the image quality of the navicular PaPr‐PaDiO view. The widely used standard beam angle of 45° appears to be the favourable angle for acquisition with a varied range of −5°. Elevation of the toe, standard in most commercially available navicular skyline cassette holders, does not influence the obtained image quality.

## Zusammenfassung

**Hintergrund:**

Palmaroproximal‐palmarodistal oblique (PaPr‐PaDiO) Röntgenbilder werden häufig zur Beurteilung des Strahlbeins (NB) angefertigt. Trotz ihrer routinemäßigen Verwendung werden unterschiedliche Aufnahmetechniken beschrieben.

**Ziele:**

Bestimmung der optimalen Hufpositionierung und des optimalen Strahlenwinkels für die Aufnahme von PaPr‐DaDiO Röntgenbildern.

**Methoden:**

Eine repräsentative Stichprobe von sechsundzwanzig Vordergliedmaßen wurde mit Hilfe einer Extremitätenpresse (um die gewichtstragende Position zu imitieren) in sechs verschiedenen Positionen fixiert. In jeder Position wurden PaPr‐PaDiO Röntgenbilder des Strahlbeins mit acht verschiedenen Strahlenwinkeln aufgenommen. Die daraus resultierenden 1248 Röntgenbilder wurden nach ihrer diagnostischen Qualität und Abgrenzung der Kompakta und Spongiosa des Strahlbeins bewertet.

**Resultate:**

Die diagnostische Qualität und die Kompakta‐Spongiosa Abgrenzung wurden bei kaudal positionierten Hufen und Winkeln zwischen 40 und 45 Grad besser bewertet. Die Erhöhung der Zehe verringerte signifikant den Winkel der Sehnengleitfläche des Strahlbeins (erhöhter Mittelwert: 40.66, SD: 4.46, nicht erhöhter Mittelwert: 42.06, SD: 4.70) (p < 0.01), schien aber keinen offensichtlichen positiven Einfluss auf die Röntgenbilder zu haben.

**Wichtigste Einschränkungen:**

Durch die Verwendung von abgesetzten Extremitäten wurden die Positionierungen nur nachgeahmt. Die Verwendung der Presse ermöglichte es die gewichtstragenden Positionen so genau wie möglich nachzubilden. Durch die Verwendung einer Zufallsstichprobe haben die Ergebnisse einen explorativen Charakter.

**Zusammenfassung:**

Eine kaudale Hufpositionierung scheint die Bildqualität der PaPr‐PaDiO‐Ansicht des Strahlbeins zu verbessern. Der weitverbreitet genutzte Strahlenwinkel von 45 Grad scheint der beste Winkel für diese Aufnahme zu sein mit einer Variation von – 5 Grad. Die Erhöhung der Zehe, die bei den meisten handelsüblichen Skyline Kassettenhaltern Standard ist, hat keinen Einfluss auf die Bildqualität.

## 1 INTRODUCTION

Routine protocols for radiographic evaluation of the navicular bone (NB) usually consist of lateromedial (LM) views, dorsoproximal‐palmarodistal oblique (DPr‐PaDiO) views and palmaroproximal‐palmarodistal oblique (PaPr‐PaDiO) views.^1^ The latter has been described with different techniques, with different positions of the limb and different angles of the primary x‐ray beam. In the first description of the PaPr‐PaDiO view, placing the foot as far caudally underneath the horse as possible was recommended.^2^ Additionally, the primary beam angle should be parallel to the palmar aspect of the digit resulting in the primary beam being in the same plane as the palmar compact bone (PB).^2^, ^3^ In a later study, a beam angle of 45° was specified but the position of the limb was not mentioned,^4^ whilst others recommended a caudal position of the foot for this beam angle.^5^ Another study used a beam angle depending on the foot conformation for the PaPr‐PaDiO view, but neither commented on foot position or beam angle nor how foot conformation influenced acquisition parameters in detail.^6^ In a recent study, a standard beam angle between 55‐65° and 35‐45° for additional views was suggested.^7^ Different angles depending on the position of the foot are described in the literature, angulation should be 45° when the foot is placed flat on the floor and an angle of 30° is recommended when a wedged block for toe elevation is used.^8^ In contrast, some authors suggest the heels should be elevated rather than the toe.^9^, ^10^ One study investigating different limb positions and primary beam angles concluded an angle of 47° was favourable, independent of the limb positions.^9^ However, in that study, a wedge block to elevate the toe was not used.

In summary, there is no universal agreement on either the beam angle or foot placement for the acquisition of the PaPr‐PaDiO view. Therefore, the aim of the current study was to investigate the optimal position of the leg and the best angle of the x‐ray beam for obtaining the PaPr‐PaDiO view. We hypothesised that a far caudal position with an elevated toe and a primary beam angle of 45° is superior to other positions and primary beam angles.

## 2 MATERIALS AND METHODS

**FIGURE 1:**
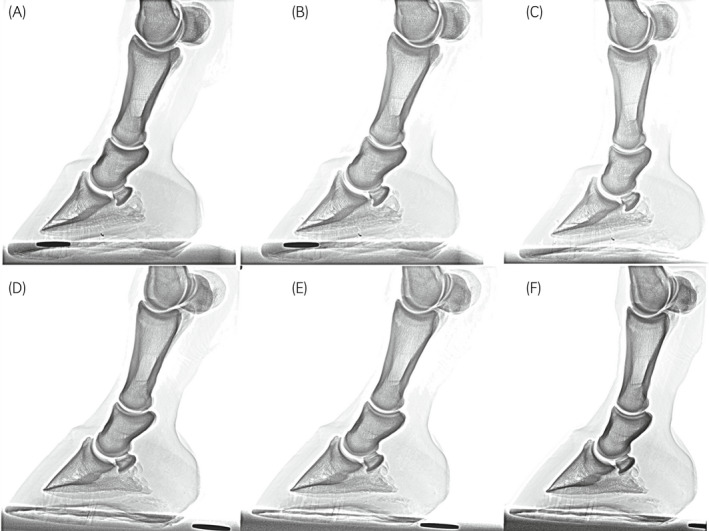
Top row (A–C) Positions without toe elevation, bottom row (D–F) positions with toe elevation. A, Position 1: Metacarpus perpendicular to the ground—without toe elevation, (B) Position 2: Foot placed 3‐cm palmar compared to position 1—without toe elevation, (C) Position 3: Foot placed 6 cm palmar compared to position 1—without toe elevation, (D) Position 4: Metacarpus perpendicular to the ground—with toe elevation, (E) Position 5: Foot placed 3‐cm palmar compared to position 4—with toe elevation, (F) Position 6: Foot placed 6‐cm palmar compared to position 4—with toe elevation

A convenience sample of 26 forelimbs was obtained from an abattoir and breed, age and reason of death were unknown. The legs were disarticulated at the middle carpal joint and a hole was drilled into the third carpal bone and the proximal end of the third metacarpal bone, to apply a custom‐made hydraulic leg press to simulate weight‐bearing positions as closely as possible. The pressure was applied to achieve full weight‐bearing of the sole (Figure 1) and to the position of the metacarpal bone perpendicular to the floor (positions 1 and 4) and with the foot 3 (positions 2 and 5) and 6 cm caudally (positions 3 and 6). To avoid artefacts, shoes were removed, feet were thoroughly cleaned and frogs were packed with commercial putty (Play‐Doh^®^, Hasbro UK Ltd).

### 2.1 Image acquisition

Palmaroproximal‐palmarodistal oblique views of all limbs were acquired with the foot positioned on a skyline block (Podoblock), in six different positions (Figure 1) using ceiling‐mounted x‐ray equipment (Siemens Optitop machine, Siemens Healthcare GmbH; Fujifilm UK Ltd) and the following palmaroproximal primary beam angles: 25°, 30°, 35°, 40°, 45°, 50°, 55° and 60°. Additionally, in all feet, lateromedial radiographs were taken in the six‐foot positions using a portable machine (Powerlight 90, Veterinary X‐Rays). This set‐up was chosen to take all the radiographs in each position without movement of the plate, foot or x‐ray machine.

### 2.2 Image rating

**FIGURE 2:**
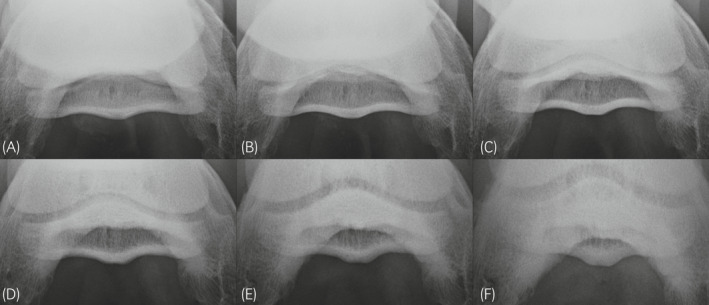
Palmaroproximal‐palmarodistal oblique views of the same limb in position 6 acquired with different primary beam angle: (A) 55°, (B) 50°, (C) 45°, (D) 40°, (E) 35° and (F) 30°. Image C was graded highest for diagnostic quality and compacta spongiosa demarcation compared to all other images. Images (A, B and D) were rated with a grade 3 for diagnostic quality, however, compacta spongiosa demarcation was lower in (A and B) compared to (D). Images (E and F) were graded lowest for diagnostic quality and compacta spongiosa demarcation

All acquired PaPrPaDiO images (n = 1248) were anonymised and each image was randomly assigned a number between 1 and 1248. The images were evaluated by a board‐certified Diagnostic Imaging Specialist and a Veterinary Surgeon unaware of the position and x‐ray beam angle used. Images were first graded for their diagnostic quality (DQ) as well as the compacta‐spongiosa demarcation (CSD) of the NB using dedicated imaging viewing software (Horos, The Horos Project) (Table 1 and Figure 2). Additionally, the sum of both criteria was calculated (SUM). For DQ the percentage of the NB visible was estimated in 25% intervals, from not visible at all to fully visible including the dorsal border. The latter should be included in an ideal radiograph to fully visualise and evaluate the navicular bone and if this is not the case an additional radiograph should be obtained.^8^ The grading system was developed and discussed by both evaluators using images from clinical cases prior to grading the study radiographs. One feature of navicular bone disease is an abnormal increase of the radiopacity of the spongiosa leading to decreased CSD. However, CSD can be artefactually decreased due to suboptimal image acquisition. The chosen ordinal scale for grading the CSD is routinely used in our clinic.

**TABLE 1:** Grading system used for the evaluation of the acquired navicular skyline views

Grade	Diagnostic quality	Compacta‐spongiosa demarcation
0	No assessment of the navicular bone is possible	Severe loss of demarcation
1	25% of the navicular bone assessable	Moderate loss of demarcation
2	50% of the navicular bone assessable	Mild loss of demarcation
3	75% of the navicular bone assessable	Well defined demarcation
4	Navicular bone and the dorsal border assessable	Not applicable

**FIGURE 3:**
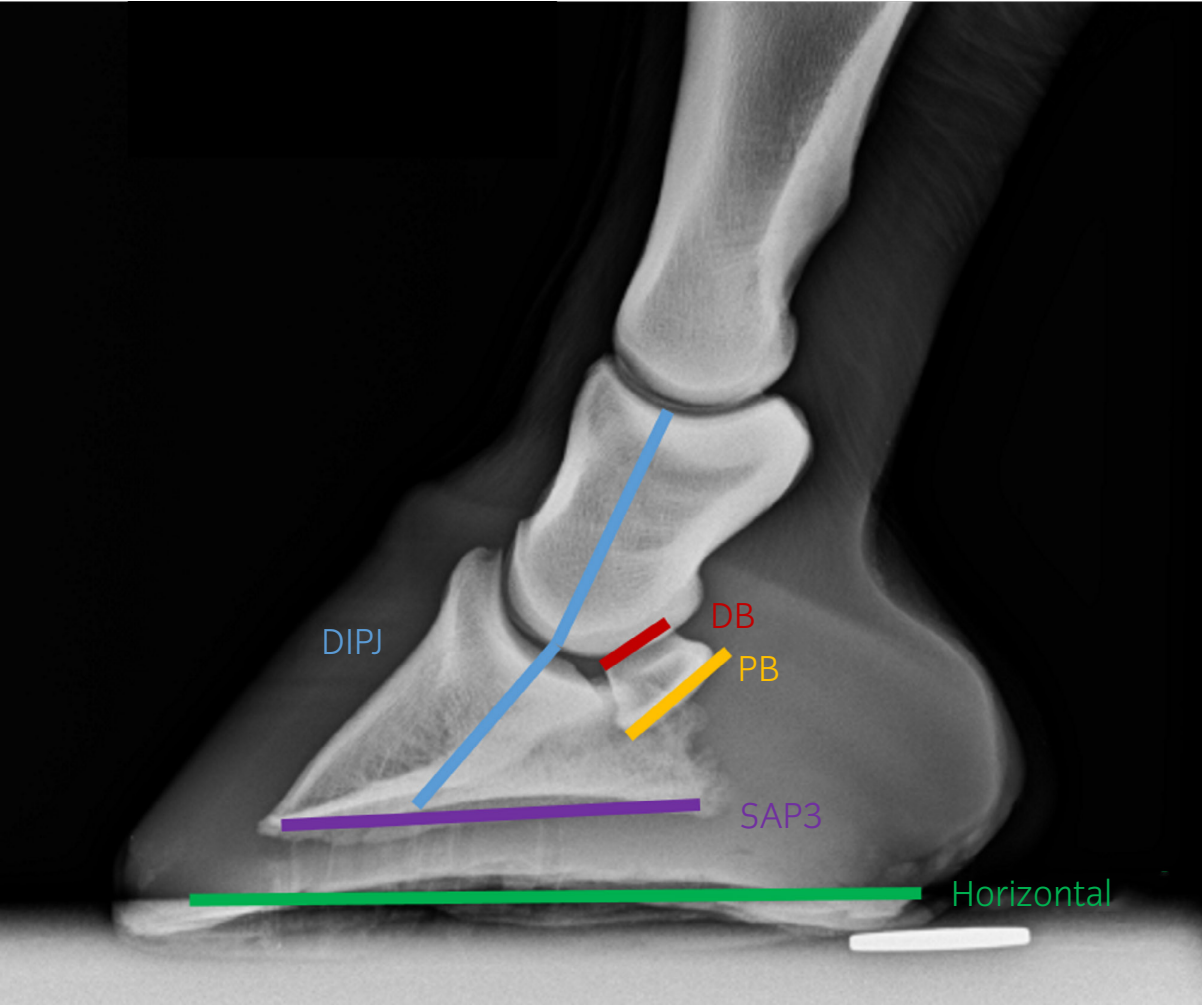
Lateromedial radiograph of the foot of one of the limbs with measurement lines for illustrative purposes: DIPJ: distal interphalangeal joint angle—The angle between the central axis of the middle phalanx and the dorsal aspect of the distal phalanx. Please note the latter is translated more palmarly. The angle of the dorsal border (DB) and palmar border (PB) of the navicular bone, as well as the solar angle of the distal phalanx (SAP3), were measured to the horizontal weight‐bearing surface

On lateromedial views, the angle of the PB and dorsal border (DB) of the NB, the solar angle of the distal phalanx (SAP3) to the ground and the angle of the distal interphalangeal joint (DIPJ) were measured^9^, ^11^ (Figure 3). Measurements of angles were repeated three times using dedicated imaging viewing software (Horos, The Horos Project) and averages were used for further statistical analysis.

### 2.3 Data analysis

Statistical analyses were performed using IBM SPSS Statistics version 26 (IBM United Kingdom Ltd). Agreement between observers was determined using weighted kappa (κ) statistics. Strength of agreement was interpreted as follows: values κ < 0.00 poor, 0.01‐0.20 slight, 0.21‐0.40 fair, 0.41‐0.60 moderate, 0.61‐0.80, substantial, 0.81‐1.00 almost perfect.^12^ Generalised estimating equation with ordinal logit link function was used for comparison of DQ, CSD and SUM in the different foot positions to position 6 and different angles of the x‐ray beam to an angle of 45°.^13^ An exchangeable matrix was used to accommodate for repeated measurements in the same leg. Data of the angle measurements were tested for normal distribution using the Shapiro‐Wilk test. For statistical analysis of the angle measurements, repeated‐measures ANOVA with Greenhouse‐Geisser post hoc tests and Bonferroni correction were used for PB and DB as the data were normally distributed. For SAP3 and DIPJ, Friedman and Wilcoxon signed‐rank tests were used as the data were not normally distributed. The significance of all statistical tests was set at *P* < .05.

## 3 RESULTS

### 3.1 Angle measurements in different positions

**FIGURE 4:**
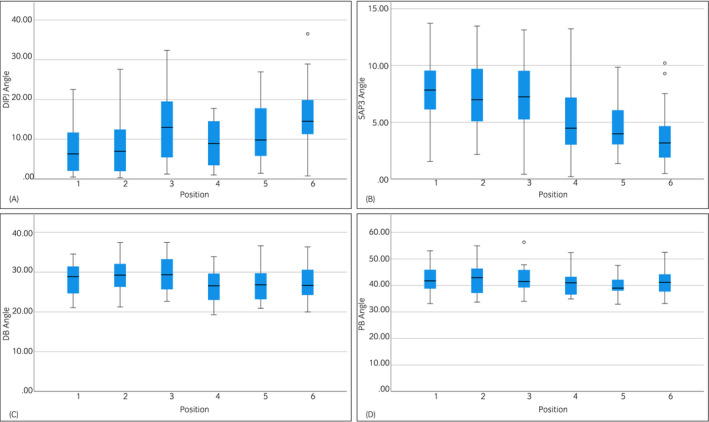
Box‐plots of the measurements of the angles on lateromedial radiographs in the different positions. DIPJ—Distal interphalangeal joint angle (A), SAP3—angle of the solar border of the distal phalanx to the weight‐bearing surface (B), DB—angle of the dorsal border of the navicular bone to the weight‐bearing surface (C). PB angle of the palmar border of the navicular bone to the weight‐bearing surface (D)

Angle measurements are presented in Figure 4. The DIPJ angle was significantly higher in position 6 compared to all other positions (*P* < .01) and significant differences were found between the other positions except between position 2 and 1, position 4 and 2 and position 3 and 5 with the more caudal positions having higher angles. The SAP3 angle was significantly different between the flat positions (positions 1, 2 and 3) and the positions with the toe elevated (positions 4, 5 and 6) (*P* < .001). No differences were found between Positions 1, 2 and 3. For the positions with the toe elevated, the SAP3 angle was smaller in position 6 compared to positions 4 and 5 (*P* < .01). The DB and PB angles were lower in positions 4, 5 and 6 compared to positions 1, 2 and 3. A significantly lower PB angle was found in position 4 compared to positions 2 and 3 (*P* < .05) as well as for position 5 to positions 1, 2 and 3 (*P* < .05). The DB angle was significantly lower in position 4 compared to positions 1, 2 and 3 (*P* < .01). In position 5, the DB angle was significantly lower than positions 2 and 3 (*P* < .05). In position 6, DB angle was lower than in position 3 (*P* < .01). No differences were found between positions 1, 2 and 3 as well as between positions 4, 5 and 6.

### 3.2 Agreement for grading between the veterinary surgeon and the diplomate

Substantial agreement between both observers was found for DQ (*κ* = 0.69, 0.95 confidence interval [CI] = 0.67‐0.72, *P* < .001) and SUM (*κ* = 0.69, CI = 0.67‐0.71; *P* < .001). For CSD, an agreement was moderate between both observers (*κ* = 0.49, CI 0.45‐0.54; *P* < .001). Overall, images were graded lower by the diplomate than by the veterinary surgeon.

### 3.3 Grading of images

**FIGURE 5:**
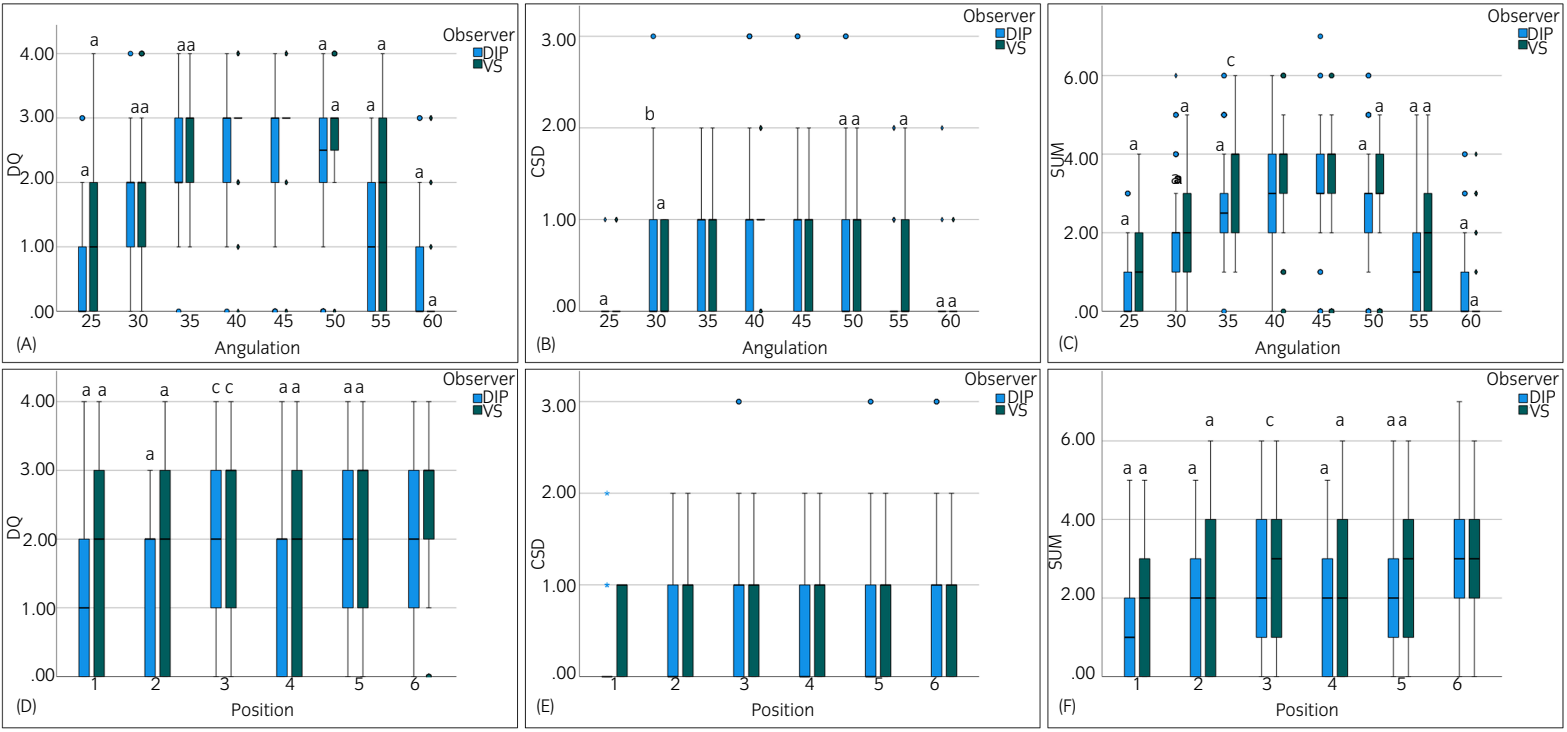
Box‐plots of the evaluation of the palmaroproximal‐palmarodistal oblique radiographs of the diplomate (DIP) and the veterinary surgeon (VS) for grading the diagnostic quality (DQ) (A, D), compacta spongiosa demarcation (CSD) (B, E) and the sum of both criteria (SUM) (C, F). Top row: angulations (A–C) and bottom row positions (D–F). Letters are indicating a significant difference of the angulation to angulation of 45° as well as a significant difference of a position to position 6. (a: *P* < .001; b: *P* < .01; c: *P* < .05)

Both observers graded the DQ of images in position 6 significantly higher than images obtained in the other positions (Figure 5). Except for images acquired with an x‐ray beam of 40°, the DQ was graded significantly higher by both observers for images obtained with an x‐ray beam angle of 45° compared to the other angles.

Compacta‐spongiosa‐demarcation was graded significantly higher for images in position 6 compared to the other positions by both observers, except for position 3. No differences were found for the grading of CSD for images obtained with the angulation of the x‐ray of 35° and 40° compared to angulation of 45°. However, grading by both observers for images acquired with the remaining angulations of the x‐ray beam was significantly lower compared to angulation of 45°.

Grading of the sum of both parameters was not different between images in positions 6 and 3 by the diplomate, but the veterinary surgeon graded images in position 6 significantly better than position 3 (*P* < .05). Grades for images acquired in all other positions were significantly lower compared to position 6 for both observers. Both observers graded images acquired with an x‐ray beam of 45° significantly higher than images with other angulations except for images obtained with an x‐ray beam angle of 40°.

## 4 DISCUSSION

Radiographs obtained with caudal foot placement with an elevated toe and x‐ray beam angle between 40° and 45° were graded higher than other positions and angulations concerning the DQ. Caudal foot placement with and without an elevated toe and x‐ray beam angles between 35° and 45° resulted in a better definition of the CSD. For the combined parameters, DQ and CSD, both observers graded images with caudal foot placement and x‐ray beam angles between 40° and 45° higher than images obtained in other positions or other x‐ray beam angulations. Despite having a substantial agreement for the combined parameter, the diplomate found no differences between images obtained either with or without toe elevation in the most caudal position; the veterinary surgeon graded the caudal positions significantly higher. Therefore, based on the results of the current study, caudal foot placement and a beam angle between 40° and 45° should be used for the acquisition of PaPrPaDiO radiographs in horses.

This beam angle is in accordance with most previous studies,^4^, ^5^, ^8^ but not used as standard in a recent study.^7^ In the latter, this was instead used as an additional angle, and the routine beam angle used was between 55° and 65°. However, angles above 55° were not found to provide high rated radiographs. Interestingly, the additional angle used in the recent study helped identify erosions of the PB.^7^ In combination with the results of the current study, using a beam angle between 40° and 45° is recommended for evaluation of the NB on PaPrPaDiO radiographs. Furthermore, these beam angles are similar to the angle of the PB measured on the lateromedial radiographs and are similar to previously published data.^9^ Therefore, these results suggest the best beam angles equal the angle of the PB, which is not surprising as for optimal radiographs the beam should be parallel to the PB.^14^

For positions with a raised toe, a flatter angle of 30° has been recommended.^8^ This conclusion is in contrast to the results of the current study, where images obtained with this beam angle were constantly graded by both observers as inferior to a beam angle of 45°. This can be explained by the measurements of the PB and DB in the LM view. These were approximately 1.9‐2.1° (DB) and 1.2‐2.1° (PB) lower in positions 4, 5 and 6 compared to positions 1, 2 and 3. Elevation of the toe by 10‐15° alters these angles marginally, therefore a different beam angle would not be justified for improved image quality.

Caudal placement of the foot was assumed to cause hyperextension of the DIPJ and, therefore, avoid superimpositions and increase the diagnostic quality.^15^ This was also found in the current study, where caudal placement increased the angle of the DIPJ, but not SAP3. Whilst toe elevation changes SAP3, it appears to have less influence on the angle of the DIPJ. The DB and PB appeared to be lower when the toe was raised, but the caudal placement of the foot did not alter these significantly.

Images obtained in positions with the metacarpal bone perpendicular to the floor were graded significantly lower than the most caudal positions, either with or without toe elevation. Therefore, acquiring radiographs in these positions might lead to insufficient DQ and an abnormal decrease in CSD. In some horses, positioning the leg caudally and on the plate might be challenging and lifting the contralateral limb will aid positioning in these cases, but will result in a position with the metacarpal bone perpendicular to the floor.^8^, ^14^ However, based on the results of the current study, the negative influence on the resulting radiograph should be considered. It appears to be more appropriate to contemplate alternative restraining methods rather than lifting the contralateral limb for sufficient image quality.

Agreement between both observers was substantial for DQ as well as the SUM and moderate for grading CSD. The latter is in accordance with previously published results, where CSD showed good intra‐ and interobserver agreement.^6^ The better agreement for DQ highlights that judging radiographs for their DQ appears to be a relatively easy task when proper guidelines are available. However, the more experienced observer graded the images lower overall; this could be due to their more critical judgement of radiographs.

The main limitation of the current study is the use of disarticulated limbs rather than obtaining the images in live horses. However, obtaining the number of views done per limb in the current study would have led to major radiation safety concerns. Furthermore, horse compliance might not have been sufficient to acquire multiple views in the same positions without movements in between. Using a custom‐made positioning aid with pressure on the limbs, weight‐bearing positions were simulated as closely as possible. Another limitation might be the dissected deep digital flexor tendon, which might have influenced the position of the limb and the NB. However, the NB has multiple ligaments to secure it in a fixed position, restricting its movement and hence the dissected deep digital flexor tendon might be neglected for influencing the position of the NB. The experimental set‐up appeared to provide sufficient stimulation of weight‐bearing conditions, with lateromedial images appearing as expected in live horses. Furthermore, on these views, the angle of the NB was within the same range as in previously published studies.^9^ Caudal positions were only obtained up to six centimetres caudally and were chosen as more caudal placement might not be possible in all live horses. Whether the further caudal placement of the limb would have led to further improved image quality was not investigated in the current study. The number of legs included was chosen at convenience, and whether a higher study population would have led to more significant differences is unknown. Finally, as no information about the orthopaedic health status of the limbs was available, it is possible that some NB were diseased and had an abnormal CSD. However, as all limbs were radiographed with the same set of image acquisition parameters and a comparison of these were made; it is presumed that this might have limited the influence of disease on the results of the current study.

The current results suggest that a beam angle between 40° and 45° should be used for the acquisition of a PaPrPaDiO radiograph of the NB to obtain an image of high DQ and optimal CSD. Caudal foot placement seems to be more beneficial for image quality than toe elevation.

## CONFLICT OF INTERESTS

No competing interests have been declared.

## AUTHOR CONTRIBUTIONS

D. Berner and M. Peeters contributed to the conceptualisation, methodology and analysis of the data. All authors contributed to the acquisition of the data. D. Berner and M. Peeters prepared the manuscript and all authors contributed to, revised and approved the final manuscript.

## ETHICAL ANIMAL RESEARCH

Ethics approval has been given by the Clinical Research and Ethical Review Board at the Royal Veterinary College (URN: M2018 0148).

## INFORMED CONSENT

Not applicable: equine samples were sourced via an abattoir.

### PEER REVIEW

The peer review history for this article is available at https://publons.com/publon/10.1111/evj.13563.

## Data Availability

The data that support the findings of this study are available from the corresponding author upon reasonable request.
